# Silicon Carbide: Material Growth, Device Processing, and Applications

**DOI:** 10.3390/ma17184571

**Published:** 2024-09-18

**Authors:** Marilena Vivona, Mike Jennings

**Affiliations:** 1 Institute for Microelectronics and Microsystems, National Research Council of Italy, 95121 Catania, Italy; 2 Centre for Integrative Semiconductor Materials (CISM), Swansea University, Swansea SA1 8EN, UK; m.r.jennings@swansea.ac.uk

The continuous demand for electronic devices operating at increasing current and power levels, as well as at high temperatures and in harsh environments, has driven research into wide-band gap (WBG) semiconductors over the last three decades. This is because, due to their outstanding physical properties, WBG semiconductors can overcome the physical and electrical limits imposed by the use of conventional silicon devices [1]. Among WBG materials, the 4H hexagonal polytype of silicon carbide (4H-SiC) is the most promising for use in power electronics applications in the medium-to-high voltage range (600–3000 V) [2,3]. However, to achieve optimized performances with these 4H-SiC devices, a full understanding of the fundamental material properties, processing technologies, and carrier transport mechanisms associated with this semiconductor material is required. In this sense, there is still plenty of room for the progress of scientific and technological research related to this material. On the one hand, an improvement to the performance of existing power devices in terms of efficiency and reliability is a key aim; on the other hand, the uses of 4H-SiC could be extended to new cutting-edge technologies, e.g., quantum technologies and sensors.

This Special Issue, entitled “Silicon Carbide: Material Growth, Device Processing, and Applications”, showcases a collection of papers on technological developments in SiC-based devices, including ten original research articles and one review paper. 

The topics addressed in the Special Issue can be categorized into three main themes: (1) investigations into the fundamental characteristics of conventional 4H-SiC devices, (2) suggestions of new approaches to developing improved devices, and (3) the use of SiC devices in emerging technology fields, such as quantum technology applications.

Among the papers focusing on important aspects of conventional 4H-SiC-based devices, methods for evaluating the reliability of the critical SiO_2_/SiC interface in planar MOSFET devices are discussed in the determination of real stress effects under extreme operational conditions [4], as well as in the assessment of the charge-to-breakdown in thermal gate SiO_2_ [5]. The effects of the various scattering mechanisms on the channel conduction are also investigated in 4H-SiC MOSFETs [6], whilethe performance of 4H-SiC MOSFETS has been evaluated using various JFET and gate oxide process parameters [7]. The inhomogeneity in Schottky barrier diodes (Pt/4H-SiC and Cr/4H-SiC contacts) has also been discussed according to the parallel-diode model and evaluated under the conditions of a wide range of operational temperatures and biases [8].

A second group of papers focuses on the issues observed during standard approaches to SiC material growth and characterization. Notably, the discoloration switching phenomenon seen during a single crystal grown of 4H-SiC [9] is discussed, as well as the direct anodic oxidation phenomenon observed on the SiC surface during conductive AFM (C-AFM) measurements in an ambient atmosphere [10].

Regarding possible advancements in SiC devices via the proposal of innovative processing solutions, a 2D material (bilayer epitaxial graphene) has been investigated and evaluated for the fabrication of new field-effect transistors on SiC [11], while a high-permittivity dielectric Al_2_O_3_/SiO_2_ stack is proposed as a gate oxide for novel devices based on the cubic polytype of SiC (3C-SiC) [12]. Additionally, new possible designs are presented for cell topologies for 4H-SiC Planar Power MOSFETs for high-frequency power applications [13].

Finally, 4H-SiC has also been discussed as an emerging material in the photonics field. A review paper dedicated to the silicon carbide on insulator stack (SiCOI) [14] provides an interesting roadmap for further developments in the use of the SiCOI key structure in quantum photonic integrated circuit applications.

Of course, due to the broadness of 4H-SiC technology, the present collection cannot provide a comprehensive presentation of all the issues. However, we are confident that fundamental properties and interesting approaches have been presented and discussed in these papers.
